# Supplementary material for: Larger colony sizes favoured the evolution of more worker castes in ants
Source: Nat Ecol Evol. 2024 Aug 26;8(10):1959–71. doi: 10.1038/s41559-024-02512-7 (PMC7616618; doi:10.1038/s41559-024-02512-7)
Supplement: Supplementary file 1 — Reporting Summary [file 41559_2024_2512_MOESM1_ESM.pdf]

nature portfolio

Corresponding author(s): Louis Bell-Roberts

Last updated by author(s): May 21, 2024

Reporting Summary

Nature Portfolio wishes to improve the reproducibility of the work that we publish. This form provides structure for consistency and transparency in reporting. For further information on Nature Portfolio policies, see our Editorial Policies and the Editorial Policy Checklist.

Statistics

For all statistical analyses, confirm that the following items are present in the figure legend, table legend, main text, or Methods section.

n/a	Confirmed	
☐	☒	The exact sample size (*n*) for each experimental group/condition, given as a discrete number and unit of measurement
☐	☒	A statement on whether measurements were taken from distinct samples or whether the same sample was measured repeatedly
☐	☒	The statistical test(s) used AND whether they are one- or two-sided<br>*Only common tests should be described solely by name; describe more complex techniques in the Methods section.*
☐	☒	A description of all covariates tested
☐	☒	A description of any assumptions or corrections, such as tests of normality and adjustment for multiple comparisons
☐	☒	A full description of the statistical parameters including central tendency (e.g. means) or other basic estimates (e.g. regression coefficient) AND variation (e.g. standard deviation) or associated estimates of uncertainty (e.g. confidence intervals)
☐	☒	For null hypothesis testing, the test statistic (e.g. *F*, *t*, *r*) with confidence intervals, effect sizes, degrees of freedom and *P* value noted<br>*Give P values as exact values whenever suitable.*
☐	☒	For Bayesian analysis, information on the choice of priors and Markov chain Monte Carlo settings
☒	☐	For hierarchical and complex designs, identification of the appropriate level for tests and full reporting of outcomes
☐	☒	Estimates of effect sizes (e.g. Cohen's *d*, Pearson's *r*), indicating how they were calculated

Our web collection on statistics for biologists contains articles on many of the points above.

Software and code

Policy information about availability of computer code

Data collection	ImageJ v1.53 used to measure ant head widths.
Data analysis	All data analysis in BayesTraits V4 or R v4.2.2. Packages used: MCMCglmm v2.34; coda v0.19-4; ape 5.7-1; dplyr v1.1.2; phylopath v1.1.3; corHMM v2.8. R code for analyses can be found at: https://github.com/LouisBell-Roberts/Larger_colony_sizes_favoured_the_evolution_of_more_worker_castes_in_ants.

For manuscripts utilizing custom algorithms or software that are central to the research but not yet described in published literature, software must be made available to editors and reviewers. We strongly encourage code deposition in a community repository (e.g. GitHub). See the Nature Portfolio guidelines for submitting code & software for further information.

Data

Policy information about availability of data

All manuscripts must include a data availability statement. This statement should provide the following information, where applicable:

- Accession codes, unique identifiers, or web links for publicly available datasets
- A description of any restrictions on data availability
- For clinical datasets or third party data, please ensure that the statement adheres to our policy

All data are provided in Supplementary Table 20 and will be made available, along with full citations of their associated references, in the public repository Dryad: https://datadryad.org/stash/share/UTQz5aoAgPXrwAJihu-Kln43Jpo30q6514MeYoB-5Xc.

Research involving human participants, their data, or biological material

Policy information about studies with human participants or human data. See also policy information about sex, gender (identity/presentation), and sexual orientation and race, ethnicity and racism.

Reporting on sex and gender	NA
Reporting on race, ethnicity, or other socially relevant groupings	NA
Population characteristics	NA
Recruitment	NA
Ethics oversight	NA

Note that full information on the approval of the study protocol must also be provided in the manuscript.

Field-specific reporting

Please select the one below that is the best fit for your research. If you are not sure, read the appropriate sections before making your selection.

☐ Life sciences ☐ Behavioural & social sciences ☒ Ecological, evolutionary & environmental sciences

For a reference copy of the document with all sections, see nature.com/documents/nr-reporting-summary-flat.pdf

Ecological, evolutionary & environmental sciences study design

All studies must disclose on these points even when the disclosure is negative.

Study description	We find strong support for the size-complexity hypothesis, with larger colony sizes appearing to favour the evolution of greater division of labour, with more worker castes and greater variation in worker size. In contrast, we did not find consistent support for alternative hypotheses for variation in division of labour being explained by either queen mating frequency or number of queens per colony.<br>Using phylogenetic comparative analyses of data on 794 species of ants from 160 different genera we show that:<br>• Colony size and queen mating frequency are both positively correlated with division of labour. However, queen number is not correlated with any other variable.<br>• We reveal a distinct evolutionary pattern where large colony sizes tend to evolve before multiple worker castes.<br>• While queen mating frequency is correlated with greater division of labour, multiple mating does not consistently evolve before the evolution of multiple worker castes.
Research sample	We analyse published data on 794 species of ants. Full details of the species studied are given in Supplementary Table 20.
Sampling strategy	We gathered data from major reviews, comparative studies and books for queen mating frequency, number of queens, number of worker castes and colony size (Blanchard & Moreau, 2017; Hughes et al., 2008; Hölldobler & Wilson, 1990; Burchill & Moreau, 2016). Data was also made available to us by the Global Ant Genomics Alliance (GAGA) consortium. We then searched published literature to retrieve all studies detailing queen mating frequency, the number of queens and the number of worker castes. We then collected available data on colony size focusing on species where data was already present for queen mating frequency or number of queens. We collected data on variation in worker size for species that we already possessed data for queen mating frequency and the number of queens. Full details on how literature was found are provided in the Methods.
Data collection	We searched published literature using the search engine Web of Science. For queen mating frequency and the number of queens we used the following key words “Ant” AND (monandr* OR monogyn* OR polyandr* OR polygyn* OR effective-mating-frequenc* OR mating-frequenc* OR paternity-frequenc* OR mating-system* OR sociogenetic-structure*). For the number of worker castes we used “Ant” AND (worker polymorph* OR worker monomorph* OR (morphometric AND worker* AND caste*) OR subcaste* OR sub-caste* OR worker dimorph* OR major-worker* OR minor-worker* OR worker AND allometr*). For colony size we used the search engine Google Scholar and searched the key words [species name] AND ‘colony size OR colony collection OR worker number’. We collected data on variation in worker size by measuring ant head widths from photos available at AntWeb.
Timing and spatial scale	Published literature were searched during 2020-21.
Data exclusions	The species excluded are supplied in Supplementary Table 21 and detailed reasons for species exclusions are presented in Supplementary Table 22. We excluded 112 species in our dataset from the analysis based on their distinct life history traits, including: (i) species that formed supercolonies (vast networks of connected nests), (ii) were social parasites, lacking some or all of the worker castes (although temporary social parasites which only establish new colonies with the assistance of a host species were included in the analysis), (iii) that can reproduce parthenogenetically to produce queens or workers, (iv) that reproduce via gamergates (mated workers that reproduce sexually), or (v) that use interlineage hybridisation for genetic caste determination. We excluded these species from the analysis as they represent secondary reductions of complexity in social organisation and are likely experiencing

different selection pressures for either the evolution of worker castes, colony size, queen mating frequency or number of queens per colony. This decision improves our ability to detect which variables could influence the evolution of the number of worker castes.

Reproducibility

All data analyses were performed in the open source software BayesTraits V4 or R v4.2.2. Packages used: MCMCglmm v2.34; coda v0.19-4; ape 5.7-1; dplyr v1.1.2; phylopath v1.1.3; corHMM v2.8. Reproducible R scripts for analyses can be found at: https://github.com/LouisBell-Roberts/Larger_colony_sizes_favoured_the_evolution_of_more_worker_castes_in_ants.

Randomization

NA. The study was a comparative analysis of published data. We did not make groups.

Blinding

NA. The study was a comparative analysis where we collected all data found in published papers for traits of interest. We analysed all species that were not excluded for the reasons detailed in the Supplementary Tables 21 and 22.

Did the study involve field work? ☐ Yes ☒ No

Reporting for specific materials, systems and methods

We require information from authors about some types of materials, experimental systems and methods used in many studies. Here, indicate whether each material, system or method listed is relevant to your study. If you are not sure if a list item applies to your research, read the appropriate section before selecting a response.

Materials & experimental systems

n/a	Involved in the study
☒	☐ Antibodies
☒	☐ Eukaryotic cell lines
☒	☐ Palaeontology and archaeology
☐	☒ Animals and other organisms
☒	☐ Clinical data
☒	☐ Dual use research of concern
☒	☐ Plants

Methods

n/a	Involved in the study
☒	☐ ChIP-seq
☒	☐ Flow cytometry
☒	☐ MRI-based neuroimaging

Animals and other research organisms

Policy information about studies involving animals; ARRIVE guidelines recommended for reporting animal research, and Sex and Gender in Research

Laboratory animals

The study did not involve laboratory animals.

Wild animals

The study involved published accounts of wild animals. The full details of the species studied are given in Supplementary table 20 and the associated references.

Reporting on sex

Findings only apply to females. All of the worker caste are female in ants.

Field-collected samples

Did not involve samples collected from the field.

Ethics oversight

No ethical approval or guidance required. The study only involved published accounts of wild animals.

Note that full information on the approval of the study protocol must also be provided in the manuscript.

Plants

Seed stocks

NA

Novel plant genotypes

NA

Authentication

NA
